# SARS-COV-2 Serological Profile in Healthcare Professionals of a Southern Italy Hospital

**DOI:** 10.3390/ijerph17249324

**Published:** 2020-12-13

**Authors:** Armando De Carlo, Sergio Lo Caputo, Carmela Paolillo, Anna Maria Rosa, Umberto D’Orsi, Maria De Palma, Pierluigi Reveglia, Donato Lacedonia, Gilda Cinnella, Maria Pia Foschino, Maurizio Margaglione, Lucia Mirabella, Teresa Antonia Santantonio, Gaetano Corso, Vitangelo Dattoli

**Affiliations:** 1 Azienda Ospedaliera-Universitaria Policlinico Riuniti di Foggia, 71122 Foggia, Italy; armdeca@gmail.com (A.D.C.); sergio.locaputo@unifg.it (S.L.C.); arosa@ospedaliriunitifoggia.it (A.M.R.); udorsi@ospedaliriunitifoggia.it (U.D.); madepalma@ospedaliriunitifoggia.it (M.D.P.); donato.lacedonia@unifg.it (D.L.); gilda.cinnella@unifg.it (G.C.); mariapia.foschino@unifg.it (M.P.F.); maurizio.margaglione@unifg.it (M.M.); lucia.mirabella@unifg.it (L.M.); teresa.santantonio@unifg.it (T.A.S.); vdattoli@ospedaliriunitifoggia.it (V.D.); 2 Dipartimento di Medicina Clinica e Sperimentale, Università di Foggia, 71122 Foggia, Italy; carmela.paolillo@unifg.it (C.P.); pierluigi.reveglia@unifg.it (P.R.); 3 Dipartimento di Scienze Mediche e Chirurgiche, Università di Foggia, 71122 Foggia, Italy

**Keywords:** SARS-CoV-2, COVID-19, asymptomatic, seroprevalence, healthcare worker, surveillance

## Abstract

Severe acute respiratory syndrome coronavirus 2 (SARS-CoV-2) is the first coronavirus that has caused a pandemic. Assessing the prevalence of anti-SARS-CoV-2 in healthcare worker groups offers a unique opportunity to study the correlation between seroconversion and immunization because of their occupational exposure and a higher risk of contagion. The study enrolled 3242 asymptomatic employees of “Policlinico Riuniti”, Foggia. After the first screening, we collected sequential serum samples for up to 23 weeks from the same subjects. In order to perform a longitudinal follow-up study and get information about the titration of IgG levels, we analyzed data from subjects (33) with at least two consecutive serological IgG—positive tests; 62 (1.9%; 95% CI: 1.4–2.3) tested positive for at least one anti-SARS-CoV-2 antibody. The seroprevalence was lower in the high-risk group 1.4% (6/428; 95% CI: 0.5–2.6) vs. the intermediate-risk group 2.0% (55/2736; 95% CI: 1.5–2.5). Overall, within eight weeks, we detected a mean reduction of –17% in IgG levels. Our data suggest a reduction of about 9.27 AU/mL every week (R^2^ = 0.35, *p* = 0.0003). This study revealed the prevalence of SARS-CoV-2 antibodies among Foggia’s hospital healthcare staff (1.9%). Moreover, the IgG level reduction suggests that the serological response fades fast in asymptomatic infections.

## 1. Introduction

Severe acute respiratory syndrome coronavirus 2 (SARS-CoV-2) is the first coronavirus that has caused a pandemic. SARS-CoV-2 was identified in the Chinese province of Hubei and rapidly spread all over the world. The World Health Organization (WHO) characterized the COVID-19 outbreak as a pandemic on 11 March 2020 [1]. As of 6 June 2020, over 6.6 million confirmed cases and 392,803 deaths worldwide had been reported [2]. Unfortunately, the current clinical management of the disease is still based on standard symptom therapies as we do not yet have vaccines or drugs that can effectively cure it.

Indeed, the COVID-19 pandemic outbreak is an unprecedented event. The rapid spread of SARS-COV-2 is most likely due to respiratory and aerial droplet transmission. However, the high number of “hidden” asymptomatic cases may also play a critical role in the transmission process. Although the diagnostic suspicion of COVID-19 was initially rooted in the presence of characteristic clinical signs and symptoms, laboratory examinations (including nasopharyngeal and oropharyngeal swab tests) have become fundamental assessments for the diagnosis of SARS-Cov-2 infection. Viral RNA is detected by using the gold standard for molecular diagnostic tests: reverse-transcription polymerase chain reaction (RT-PCR) and/or real-time RT-PCR (rRT-PCR). Unfortunately, the method’s predictive rates are influenced by viral titer, incorrect sample collection, transportation, storage, and sample type [3,4]. In a recent study, the positive rate was reported at 93% in bronchoalveolar lavage samples, while it was only 32% in pharyngeal swabs [3]. Moreover, RT-PCR test accuracy and repeatability changed within the time-window it was being performed. Viral RNA can be detected as early as one day after symptoms, but test positivity starts to decrease within three weeks until the virus becomes undetectable. There is evidence showing an association between disease severity, viral load, and how long the viral RNA can be detected from the onset of illness.

The false-negative RT-PCR gap, mostly false-negatives of asymptomatic but still contagious individuals, can be problematic during a pandemic. From this perspective, the detection of the anti-SARS-CoV-2 antibody can enhance COVID-19 diagnostic sensitivity. Furthermore, testing the host’s immune response to SARS-CoV-2 infection may be critical for patients with mild-to-moderate disease, who are often clinically observed only after two weeks of illness onset. Total antibodies measurement is the most sensitive serological marker, with an increase in the detectable level around the second week from symptom onset [5]. However, the available SARS-CoV-2 assays utilize mainly IgG and/or IgM, total immunoglobulins, and in some cases, IgA.

Serological evaluation is essential for investigating the extent of COVID-19 in the general population and evaluating the potential effectiveness of serum antibodies as a protective factor against future disease. Moreover, assessing the prevalence of anti-SARS-CoV-2 in healthcare worker groups offers a unique opportunity to study the correlation between seroconversion and immunization because of their occupational exposure and a higher risk of contagion [6,7]. Finally, the surveillance results among asymptomatic health workers are also beneficial to mitigate workforce depletion due to unnecessary quarantine, to identify atypical, mild, or asymptomatic cases, and to protect all healthcare workers. The shortage of staff in healthcare is representative of the global effort against coronavirus disease 2019.

We conducted a seroprevalence study to evaluate the levels of anti-SARS-CoV-2 antibodies in samples taken from over 3000 healthcare workers in our hospital, “Policlinico Riuniti” of Foggia. We aim to determine the prevalence of SARS-CoV-2 antibodies, seroconversion, and IgG persistence in asymptomatic healthcare workers.

## 2. Materials and Methods

### 2.1. Samples

In agreement with the Department of Health Promotion of the Apulia region, the Chief of Staff of our hospital activated a strategy for early detection of potential COVID-19 outbreaks among the personnel of Policlinico Riuniti Hospital, Foggia. This project has been approved by the “Comitato Etico Area 1” Prot. n. 152/C.E/2020. The first step of surveillance was a serological investigation of hospital healthcare workers. Written informed consent was obtained from each enrolled subject. From 17 March to 18 May 2020, we enrolled a total of 3242 employees (age 46.5 ± 11.7 years), all with no SARS-CoV-2 symptoms (Table 1) and no history of previous positive testing. Symptoms (respiratory symptoms or flu-like symptoms or fever above 37.5 °C) were assessed by self-report at the beginning of the enrollment and follow-up time. Since this is a retrospective study, individuals that developed symptoms during the enrollment and/or follow-up were not taken into account. A control group (Pre-COVID-19) of 83 samples of sera from another unrelated study [8,9] was also tested. The samples were collected before the Italian COVID-19 outbreak (2015–2017). The employees’ group was stratified into 3 subgroups, as follows: (1) Departments with a higher potential risk of contracting SARS-CoV-2, such as the emergency room (ER), intensive care unit (ICU), and pneumology unit (PI), infectious diseases (ID), and laboratory staff; (2) the remaining departments were classified as an intermediate-risk group (Other Departments); (3) administrative personnel (Smart Working Offices), with a lower risk of contracting SARS-CoV-2. Positive results to either IgG or IgM were reported to the attending physician. Moreover, nasopharyngeal swabs and SARS-CoV-2 nucleic tests were performed on all seropositive individuals (Allplex™ 2019 n-CoV Assay, Seegene, Seoul, Korea).

The second step was to perform a longitudinal follow-up serological test to study the variation of IgG levels in the studied subjects. We collected sequential serum samples for up to 23 weeks after the first test. At the time of the drafting of this manuscript (1 September 2020), 33 subjects had had at least two consecutive serological IgG positive tests and were included in our analysis.

### 2.2. Methods

We used a chemiluminescent immunoassay (Shenzhen YHLO Biotech, Shenzhen, China) to study the seroprevalence of SARS-COV-2-specific antibodies (IgG and IgM against nucleocapsid and spike proteins). The assay was performed according to the manufacturer’s instructions on an iFlash1800 immunoassay analyzer (Shenzhen YHLO Biotech, Shenzhen, China), which automatically calculates the amount of anti-SARS-CoV-2 antibodies (2 separate kits for IgG and IgM) that is correlated with the relative light units (RLUs) resulting from the chemiluminescent reaction. The concentrations (AU/mL) were determined via an instrument-specific calibration curve. Following intralaboratory validation, we set a cut-off of 8 AU/mL for both IgG and IgM, with a specificity of 98.8% and 100% and sensitivity of 97% and 78%, respectively. Precision was evaluated by measuring daily internal quality controls (IQCs) and a human serum pool of samples with different concentration values of IgG and IgM antibodies (IgG: 4.89 to 17.16 AU/mL (CV% 4.78–5.87), IgM: 3.86 to 16.27 AU/mL (CV% 5.6–5.49), lower–upper levels, respectively). Nasopharyngeal swabs were performed on healthcare workers with IgG and IgM values over the cut-off of 8 AU/mL, as well as between 6 and 7.9 AU/mL.

### 2.3. Statistic

All continuous characteristics are shown as the mean (±SD), and categorical characteristics are shown as numbers (*n*) or percentages (%). One-way ANOVA was used to analyze the average RLU value difference between the different participant groups; *p*-value < 0.05 indicated significance and 95% confidence intervals (95% CIs) of population estimates were calculated by performing bootstrap resampling. All box plot graphs represent the minimum, the maximum, the sample median, and the first and third quartiles. Statistical analyses were performed in R v3.6.3 (RStudio, Boston, MA, USA) and GraphPad Prism (version 7.0.4, GraphPad Software, San Diego, CA, USA).

## 3. Results

### 3.1. Seroprevalence

**Figure 1:**
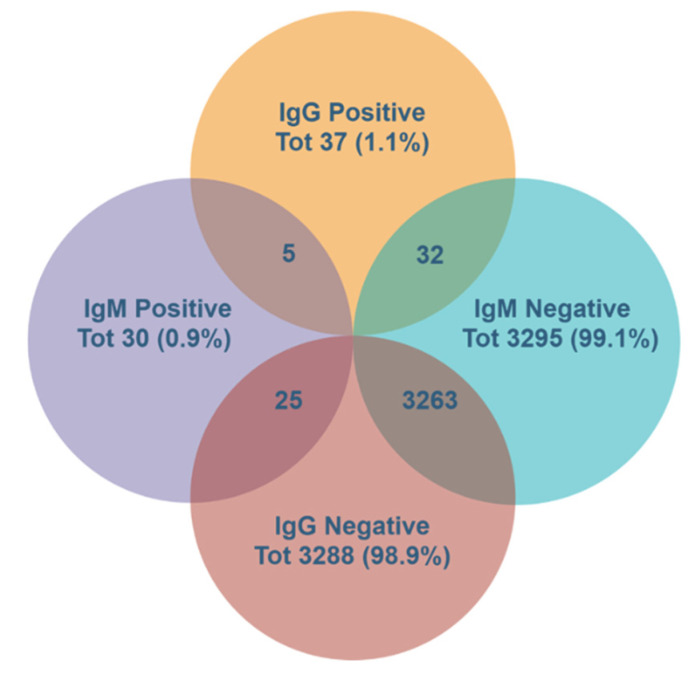
Serological diagnosis of severe acute respiratory syndrome coronavirus 2 (SARS-CoV-2). Summary of IgG and IgM results among the healthcare workers.

Overall, 62 individuals (1.9%) tested positive for at least one anti-SARS-CoV-2 antibody among the employees’ group. Of these 62, only five individuals (8.0% of the positive group, 0.15% of the employees’ group) had both IgG and IgM positive test results (double-positive), while 32 and 25 had only IgG and only IgM positive results, respectively (Figure 1).

**Figure 2:**
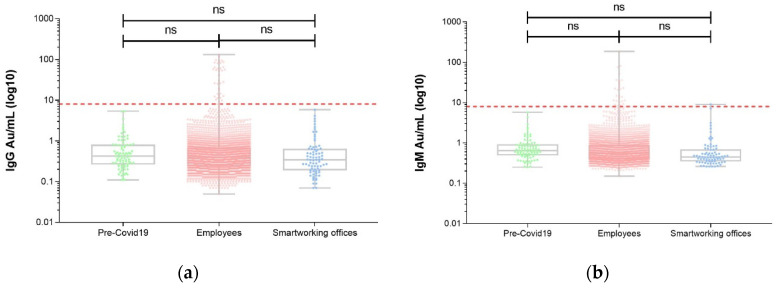
Overall seroprevalence trends at Foggia Hospital “Policlinico Riuniti” during COVID-19 outbreak and lockdown (17 March to 18 May 2020). General IgG (**a**) and IgM (**b**) seroprevalence in 3164 healthcare workers, 78 administrative personnel (Smart Working Offices) enrolled during our COVID-19 study, and 83 plasma samples collected before the COVID-19 outbreak in Italy (2015–2017). Ig values are expressed as the original concentration (AU/mL). Cut-off was set at 8 AU/mL. One-way ANOVA: (**a**) *p* = 0.654; (**b**) *p* = 0.840. ns: non significant.

All the Pre-COVID-19 samples tested negative for both IgG and IgM. In order to better analyze the seroprevalence trends at Foggia Hospital during the COVID-19 outbreak and lockdown, we compared the Pre-COVID-19 group′s IgG and IgM levels to the Smart Working Offices group and the remaining employees (Figure 2).

**Figure 3:**
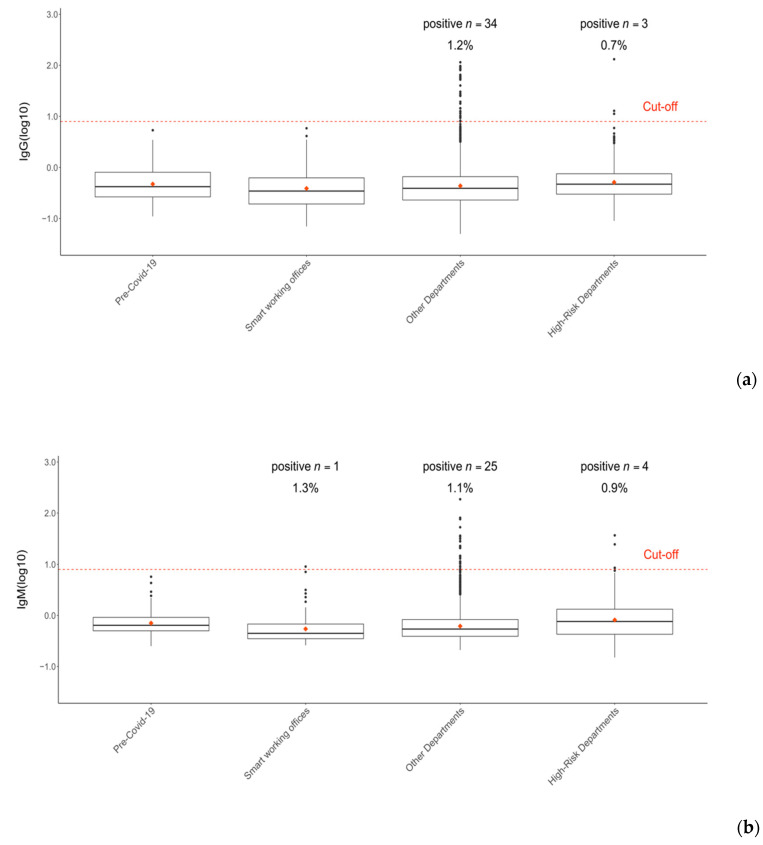
Stratified seroprevalence trends at Foggia Hospital” “Policlinico Riuniti” during the COVID-19 outbreak and lockdown (17 March to 18 May 2020). IgG (**a**) and IgM (**b**) seroprevalence of 3242 hospital employees stratified by departments. Ig values are expressed as log_10_ of the original concentration (AU/mL). Cut-off was set at 8 AU/mL (log_10_(8) = 0.9).

IgG levels for 38 employees were over the cut-off point. We detected positive IgM levels in 29 healthcare workers and one individual of the Smart Working Offices group. All 62 positive subjects were also tested for the presence of SARS-CoV-2 nucleic acid. Viral RNA was detected in nine individuals (13.8% of Ig-positive group) by RT-PCR. Nasopharyngeal swabs were also performed on nine healthcare workers with IgG or IgM concentrations between 6 and 7.9 AU/mL. All of them tested negative for the presence of viral RNA. The employees’ group was further stratified into three subgroups, as previously described in the sample section. The fraction of healthcare workers that tested positive among the employees’ group varied from 0.7% to 1.3% when considering IgG and IgM separately. The proportion of positive subjects in the high-risk group was 0.7% and 0.9% for IgG and IgM, respectively (Figure 3).

Surprisingly, a higher proportion was detected in the intermediate-risk group (IgG 1.2%, IgM 1.1%) and the low-risk group (IgM 1.3%). Instead, the cumulative proportion of individuals who tested positive (IgG and/or IgM) varied between 1–2.4% (ER = 1%, ICU = 2%, other departments = 2.1%, pneumology unit = 2.2%, and laboratory = 2.4%). The average level of IgG and IgM antibodies of each subgroup is summarized in Table 1.

**Figure 4:**
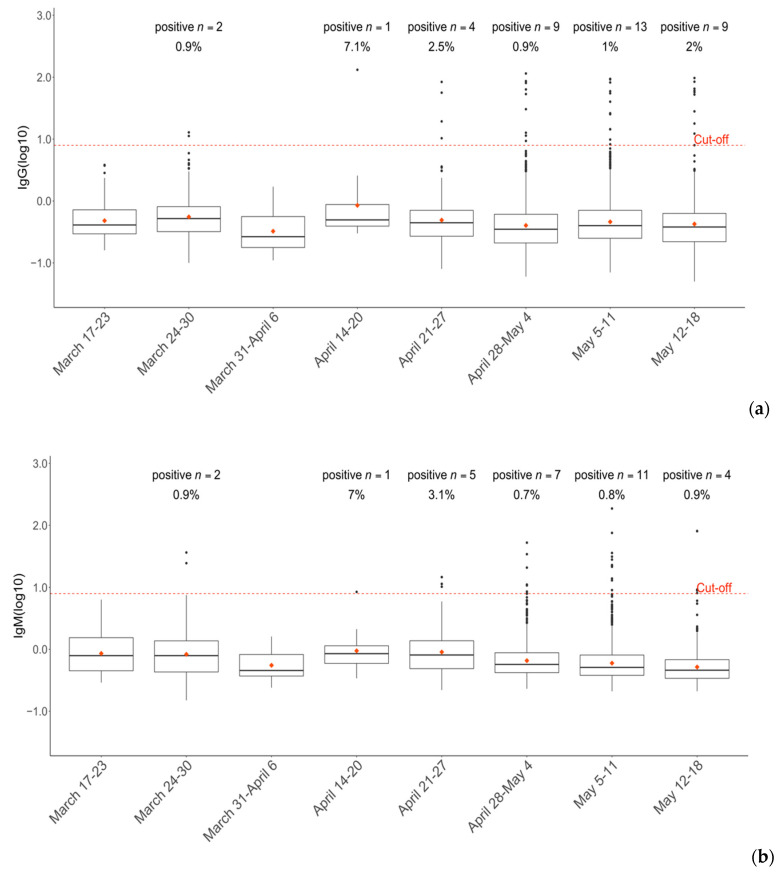

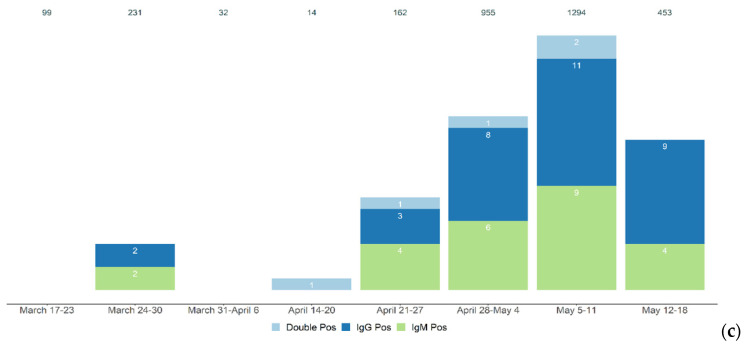
Time-lapse of healthcare seropositivity at Foggia Hospital” “Policlinico Riuniti” during the COVID-19 outbreak and lockdown (17 March to 18 May 2020). IgG (**a**) and IgM (**b**) seroprevalence stratified by weekly time points. Ig values are expressed as log10 of the original concentration (AU/mL). Cut-off was set at 8 AU/mL (log_10_(8) = 0.9). (**c**) Number of COVID-19-positive healthcare workers by weeks of enrollment.

We investigated the prevalence of SARS-CoV-2 antibodies in our hospital community across the nine weeks of enrollment (17 March to 18 May 2020). A higher fraction of positive results (2.5% IgG, 3.1% IgM) was detected during the 6th week of our enrollment (21–27 April). In that week, 163 individuals were evaluated and 8 tested positive for the SARS-CoV-2 antibody (4.9%). Figure 4 summarizes the number of positive Ig tests (IgG 4a, IgM 4b, respectively) and the overall number of COVID-19-positive healthcare workers (4c), stratified by week. During the 4th week of enrolment (7–13 April), no tests were performed.

### 3.2. IgG Titration

**Figure 5:**
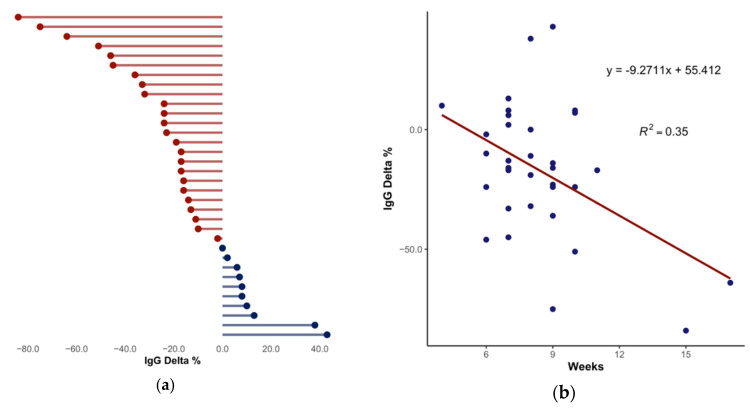
Longitudinal study. (**a**) IgG level variation in 33 asymptomatic subjects. (**b**) Negative correlation between IgG Delta % and elapsed weeks (one-way ANOVA, *p* = 0.0003). In total, 67% of subjects (*n* = 22) showed a decrease in IgG levels.

Finally, we investigated the persistence of IgG levels in our selected asymptomatic population. We collected and analyzed data from sequential serological testing. In order to study the variation in IgG levels in time, only individuals (*n* = 33) with two consecutive positive serological samples were included in this analysis. With an average of about eight weeks, the two samples’ elapsed time varied from 4 to 17 weeks. IgG average concentrations were 44.78 and 36.42 AU/mL, with an average delta (second sample–first sample) of −7.42 AU/mL (−17%, mean percentage of decrease). Among the 33 subjects, only 8 showed an increase in IgG levels (between 6% and 43%). IgG levels in 22 subjects declined (range of percentage of decrease was between –6% and –84%), while the remaining 3 showed a steady level of IgG (between −2% and 2%; Figure 5a). Figure 5b shows the negative correlation between IgG percentage of decrease (IgG delta %) and the weeks elapsed (R^2^ = 0.35, *p* = 0.0003). Our data suggest a reduction of about 9.27% in IgG level every week. None of the subjects became seronegative during the enrollment and follow-up periods.

## 4. Discussion

SARS-CoV-2 serological surveillance can assess the presence of antibodies in serum or plasma and their efficacy as a protective factor against disease in future contact (if neutralizing). Furthermore, the seropositive rate provides information on the prevalence of COVID-19 in the general population. This systematic study aimed to determine the prevalence of SARS-CoV-2 antibodies and the seroconversion among the healthcare workers within our hospital. Healthcare staff surveillance is crucial during this pandemic—it helps reduce potential healthcare-associated transmission by early disease detection. IgG and/or IgM antibodies were detected in 62 of 3242 (1.9%; 95% CI: 1.4–2.3) employees (61 health care workers and 1 administrative personnel). Asymptomatic and symptomatic patients can exhibit different kinetics of IgG/IgM responses to SARS-CoV-2. In our study, we identified 25 IgM+ IgG− individuals. These data suggest that IgM testing on asymptomatic populations could suffer from a high false-positive rate and has to be interpreted with precaution. It is important to remember that serological testing is not used for COVID-19 diagnoses. Being IgM seropositive could suggest recent contact with SARS-CoV-2, but this has to be confirmed by molecular testing. The seroprevalence was lower in the high-risk group (1.4%, 6/428; 95% CI: 0.5–2.6) vs. the intermediate-risk group (2.0%, 55/2736; 95% CI: 1.5–2.5). Only one participant (1.3%, 95% CI: 0–3.8) of the low-risk group tested positive for SARS-CoV-2 IgM antibodies. Therefore, the cumulative seroprevalence among the employees was low (1.9%; 95% CI: 1.4–2.4), which is aligned with other recent SARS-CoV-2 seroprevalence studies [10,11,12,13]. One of these studies was conducted on a total of 17,368 individuals in China, where the virus was first observed. The seropositive prevalence rate in the subcohort of healthcare workers (*n* = 714) from the city of Wuhan (the epicenter of the COVID-19 pandemic) was 3.8%. In contrast, the seropositive prevalence rate went down to 1.3% among the subcohort of healthcare workers (*n* = 3091) from two nearby cities of the Hubei province and to 1.2% among 260 healthcare workers from two other cities farther south of Wuhan [10]. The differences in seropositive rates in different geographic areas were consistent with China’s SARS-CoV-2 spreading pattern. Similar considerations as the Chinese seroprevalence geographical distribution can also be made for Italian seropositive rates. Indeed, seroprevalence trends in healthy blood donors from Milan (the Italian epicenter of the COVID-19 pandemic) were estimated to be as high as 4.6% in the last week of February 2020, with an observed rate of increase in IgG+ of 2.7% ± 1.3% per week [11]. Moreover, the reported positive COVID cases on 10 June 2020 were 905.6 and 112 for every 10,000 inhabitants in the Lombardy and Apulia regions, respectively.

Two Germans studies also investigated the seroprevalence for anti-SARS-CoV-2 antibodies among healthcare workers [12,13]. The first study was conducted among 316 healthcare workers of the University Hospital Essen, Germany. IgG antibody’s seroprevalence was 1.6% during the weeks between 25 March and 21 April 2020 [12]. The COVID-19 Contact (CoCo) study also reported anti-SARS-CoV-2 IgG prevalence in the range of 1–2% among health care workers [13]. The general population’s seropositive rate was estimated in a study conducted in Santa Clara County (USA). Approximately 3500 residents within 15 miles radius of the testing site were enrolled. The seroprevalence among the selected population (weighted for demographic and income distribution) was estimated at 4.65% [14].

In our study, viral RNA was detected in only nine individuals (13.8% of 62 Ig-positive cases) by RT-PCR. These data are consistent with previous findings and emphasize the benefit of serological investigation among the asymptomatic population, principally when combined with molecular tests. In our region, the number of positive results to RT-PCR was low (112 per 10,000 inhabitants; 1.12%) compared to those in the north of Italy. Given these data, our study also pointed out a low seropositivity rate (1.9%) among healthcare workers (a population with a potentially higher risk of contagion). Such a value is far from herd immunity. However, if we apply the 1.9% rate to the healthy population of Foggia county (631,000 inhabitants), we should have had about 12,000 COVID-19-positive cases. In contrast, the reported cases in our county on 10 June 2020 were only 1162. The reported cases are most likely underestimated, considering that the asymptomatic population is often not identified by clinical and/or molecular laboratory tests. Our preliminary findings estimated that the combination of serological screening with molecular testing could identify an additional 0.78% of the asymptomatic population. Finally, the persistence of IgG antibody responses in our cohort of asymptomatic subjects (33) was low. Only eight subjects showed an increase in IgG levels between the first and second samples; three others had steady IgG levels, and the remaining 67% of subjects showed a decrease in IgG levels. These observations are related to a limited number of asymptomatic subjects (33) and need to be confirmed in a more significant cohort. A decrease in IgG levels after eight weeks has also been reported in another small cohort of the asymptomatic population [15]. In this study, 37 asymptomatic subjects were tested eight weeks apart and showed a decreased median percentage of 71.1% (range, 32.8–88.8%) [15]. These findings agree with the results published by Zhao et al. (2020) [16]. Indeed, Zhao’s study showed that the IgG levels of the asymptomatic group decreased below the cut-off value of 10 AU/mL by week 7 [16].

## 5. Conclusions

This surveillance study reveals the prevalence of SARS-CoV-2 antibodies among Foggia’s hospital asymptomatic healthcare employees (1.9%). Moreover, the data suggest a serological response that fades fast in asymptomatic subjects. Additional longitudinal serological studies that profile asymptomatic individuals are needed to determine the persistence of antibody-mediated immunity.

## Figures and Tables

**Table 1:** Demographics and serological data of enrolled Policlinico Riuniti healthcare workers and the control group.

		Smart Working Offices	Other Departments	ER	ICU	Pneumology Unit	Infectious Diseases	Laboratory	Total Employees	Pre-COVID-19
No. (%)		78 (2.2)	2736 (84.4)	96 (2.9)	100 (3)	90 (2.7)	57 (1.7)	85 (2.6)	3242	83
Age	Mean (±SD)	51.3 (9.1)	46.7 (11.6)	43.1 (12.3)	38.8 (9.6)	43.7 (12.6)	41.6 (14.6)	48.9 (10.2)	46.5 (11.7)	49 (14.8)
IgG, AU/mL	Mean (±SD)	0.68 (0.98)	1.28 (6.96)	0.62 (0.66)	0.78 (1.27)	0.86 (1.51)	0.74 (0.72)	2.27 (14.13)	1.14 (6.12)	0.72 (0.92)
	Min–Max	0.07–5.85	0.05–131.00	0.11–4.7	0.10–11.22	0.11–12.84	0.13–3.73	0.09–131.62	0.05–131.62	0.11–5.36
	Positive tests	0	34	0	1	1	0	1	37	0
IgM, AU/mL	Mean (±SD)	0.80 (1.30)	1.07 (4.79)	1.36 (3.74)	1.39 (1.47)	1.14 (2.67)	1.23 (1.20)	1.06 (1.13)	1.04 (4.31)	0.88 (0.82)
	Min–Max	0.26–9.04	0.16–186.39	0.22–36.59	0.22–8.57	0.15–24.62	0.26–6.36	0.22–8.46	0.15–186.39	0.25–5.72
	Positive tests	1	25	1	1	1	0	1	30	0
Positive subjects		1	55	1	2	2	0	1	62	0

SD: Standard Deviation.
